# ATHLOS Healthy Aging Scale score as the predictor of all-cause mortality in Poland and Czechia

**DOI:** 10.3389/fpubh.2023.1114497

**Published:** 2023-03-16

**Authors:** Magdalena Kozela, Andrzej Pająk, Krystyna Szafraniec, José Luis Ayuso-Mateos, Martin Bobak, Wentian Lu, Hynek Pikhart, Maciej Polak, Albert Sanchez-Niubo, Urszula Stepaniak, Josep Maria Haro

**Affiliations:** ^1^Department of Epidemiology and Population Studies, Jagiellonian University Medical College, Krakow, Poland; ^2^Centro de Investigación Biomédica en Red de Salud Mental (CIBERSAM), Madrid, Spain; ^3^Department of Psychiatry, Universidad Autónoma de Madrid, Madrid, Spain; ^4^Department of Epidemiology and Public Health, University College London, London, United Kingdom; ^5^Research Centre for Toxic Compounds in the Environment (RECETOX), Masaryk University, Brno, Czechia; ^6^Department of Social Psychology and Quantitative Psychology, University of Barcelona, Barcelona, Spain; ^7^Research, Innovation and Teaching Unit, Parc Sanitari Sant Joan de Déu, Sant Boi de Llobregat, Spain

**Keywords:** healthy aging, scale, mortality, Central and Eastern Europe (CEE), aging

## Abstract

**Background:**

The ATHLOS consortium (Aging Trajectories of Health–Longitudinal Opportunities and Synergies) used data from several aging cohorts to develop a novel scale measuring healthy aging comprehensively and globally (ATHLOS Healthy Aging Scale). In the present study, we assessed the predictive performance of the ATHLOS Healthy Aging Scale for all-cause mortality in middle-aged and older adults.

**Methods:**

Data from the Polish and Czech HAPIEE (Health Alcohol and Psychosocial factors In Eastern Europe) prospective cohorts were used. There were 10,728 Poles and 8,857 Czechs recruited. The ATHLOS Healthy Aging Scale score was calculated for all participants using data from the baseline examination carried out from 2002 to 2005. The follow-up for all-cause mortality was completed over 14 years. The associations between quintiles of the ATHLOS Healthy Aging Scale and all-cause mortality were estimated using Cox proportional hazards models.

**Results:**

A total of 9,922 Polish and 8,518 Czech participants contributed ATHLOS Healthy Aging Scale and mortality data with 1,828 and 1,700 deaths, respectively. After controlling for age, the ATHLOS Healthy Aging Scale score was strongly associated with mortality in a graded fashion for both genders and countries (hazard ratios for lowest vs. highest quintile were 2.98 and 1.96 for Czech and Polish women and 2.83 and 2.66 for Czech and Polish men, respectively). The associations were only modestly attenuated by controlling for education, economic activity, and smoking, and there was further modest attenuation after additional adjustment for self-rated health.

**Conclusion:**

The novel ATHLOS Healthy Aging Scale is a good predictor of all-cause mortality in Central European urban populations, suggesting that this comprehensive measure is a useful tool for the assessment of the future health trajectories of older persons.

## 1. Introduction

Populations around the world are aging faster than ever before. The ongoing process is the result of the favorable phenomenon of increasing life expectancy and is exacerbated by the low fertility rate. Achieving healthy aging is an important challenge worldwide. Many initiatives have attempted to operationalize the concepts of healthy aging (1, 2). However, international consensus regarding how healthy aging should be measured, while acknowledging the diversity between populations, has not been fully achieved (3–5).

In 2015, the World Health Organization (WHO) defined healthy aging as “the process of developing and maintaining the functional ability that enables well-being in older age” (6). Functional abilities are health-related attributes that allow people to do what they have reason to value. They can be determined by intrinsic capacities (the composite of all the physical and mental capacities that an individual can draw on) and social environment, as well as the interactions between them. The WHO suggested that building and maintaining intrinsic capacity is the fundamental way of enhancing functional ability. Agreement on metrics, measures and analytical approaches to healthy aging was recognized as an urgent need (6). This has led to attempts to develop common metrics of healthy aging that would allow for comprehensive comparisons of healthy aging profiles globally (6). Developing common metrics of healthy aging would also facilitate cross-country analyses of healthy aging and its determinants within the different societal contexts of older adults (4).

The ATHLOS (Aging Trajectories of Health–Longitudinal Opportunities and Synergies) consortium (7) harmonized aging cohorts worldwide and developed a novel scale to measure healthy aging comprehensively and globally. The ATHLOS Healthy Aging Scale was constructed based on 41 characteristics referring to intrinsic capacity and functional ability (6) using integrated data from over 411,000 individuals from 16 independent aging cohorts, covering 38 countries from all continental regions, which has been believed to be universally applicable for evaluating healthy aging at an individual level (8).

A well-designed index of healthy aging should reflect an individual's biological age and predict mortality independently of calendar age. So far, the ATHLOS Healthy Aging Scale has been found to be inversely and progressively related to 10-year all-cause mortality across six waves of data collection in a sample of nearly 11,000 participants from England (9). This study also found that the older the participants, the stronger the protective effect of the higher scores from the ATHLOS Healthy Aging Scale observed. As the performance of the ATHLOS Healthy Aging Scale may differ across populations, potentially reflecting different stages of social, demographic, and epidemiological transition, more analyses of the scale should be performed in diverse population samples to assess the predictive ability in terms of all-cause mortality.

In this context, Central and Eastern Europe provide interesting settings for a study of healthy aging. In the 1990s and early 2000s, Central and Eastern European countries were experiencing political and economic transitions. After a long period of stagnating or falling life expectancy, which led to a large mortality gap between Eastern and Western Europe, life expectancy has increased dynamically (10).

In this report, we used two Central European prospective population-based cohorts to assess the relationships between the ATHLOS Healthy Aging Scale and all-cause mortality in middle-aged and older adults.

## 2. Methods

### 2.1. Data

The Polish and Czech cohorts of the Health, Alcohol and Psychosocial factors In Eastern Europe (HAPIEE project) were established in Krakow (Poland) and six Czech towns between 2002 and 2005 (11). Both cohorts included random samples of men and women aged 45–69 years at baseline, stratified by gender and 5-year age groups. In total, we recruited 10,728 Poles and 8,857 Czechs (the response rate was 61% in Poland and 55% in Czechia). All participants gave written consent. At baseline, participants were interviewed by trained nurses using a standardized questionnaire. Detailed information on health, including physical functioning assessment and mental health and cognitive functioning tests, was collected. Information on education, marital status, occupational status, smoking, and self-rated health was also obtained.

### 2.2. Mortality information

The Czech cohort was followed up for mortality until 31 December 2018. In Poland, the follow-up was completed by 25 August 2017. In Poland, mortality data from the Central Registry of Residents and the Central Statistical Office were used. In the Czechia, mortality data from the National Death Register was used.

### 2.3. ATHLOS Healthy Aging Scale

The ATHLOS Healthy Aging Scale was constructed using harmonized data from 16 international cohorts. The harmonization aimed to convert study-specific variables into a priori defined variables and their possible values to provide the same variables format across studies. A list of 41 characteristics referring to intrinsic capacity and functional ability assessed in the studies, covered domains such as vitality, sensory skills, locomotion/mobility, cognition and activities and instrumental activities of daily living. The ATHLOS Healthy Aging Scale was constructed using a two-parameter logistic item response theory model with characteristics related to intrinsic capacity and functional ability, and heterogeneities in the cohort-specific datasets. The item response theory approach targets the explanation of the relationship between latent traits and their manifestations, by establishing a link between the individuals' responses to specific items and the underlying trait, on an assumed continuum. This approach aims at the assessment of the individual's position on the continuum scale (8, 12). The IRT models presented high reliability (>0.90) (8). The obtained score is normally distributed with a mean of 50 and a standard deviation of 10, with higher values indicating better health (8). The ATHLOS Healthy Aging Scale scores were made for all ATHLOS individuals, including the HAPIEE cohorts, and the estimation took into account non-responses and imputed missing data. The harmonization algorithms of each item per study can be found at URL: https://github.com/athlosproject/athlos-project.github.io. A detailed description of the harmonization procedure and the delivery of the ATHLOS Healthy Aging Scale have been published by Sanchez-Niubo et al. (7, 8).

### 2.4. Covariates

Based on a previous study (9), age (continuous), gender (binary: men vs. women), marital status (binary: married/cohabiting vs. single/widowed/divorced), education (binary: university vs. lower), occupational status (binary: employed vs. not in work), smoking (binary: ever-smoker vs. never-smoker), and self-rated health (binary: good vs. lower) were considered as potential confounding factors.

### 2.5. Statistical analyses

All HAPIEE cohort participants who provided consent for mortality follow-up and had complete data for the ATHLOS Healthy Aging Scale and covariates were included in statistical analyses (*N* = 9,922 in Poland and 8,518 in Czechia; 94% of the full sample). The distributions of the ATHLOS Healthy Aging Scale and other covariates in men and women were examined separately in each country. Cohorts were divided into five subgroups according to gender-specific quintiles of the ATHLOS Healthy Aging Scale, assessed for the countries combined. The ranges of quintiles for women were: Q1 ≤ 40.11; Q2:40.12–45.68; Q3:45.69–51.66; Q4:51.67–57.86; Q5 > 57.86 and for men were: Q1 ≤ 43.07; Q2:43.08–50.08; Q3:50.09–55.98; Q4:55.99–60.06; Q5 > 60.06. The reference category was the highest quintile (P5).

Firstly, country differences in sample characteristics at baseline and follow-up time were examined in men and women separately. Secondly, the associations between ATHLOS Healthy Aging Scale scores and all-cause mortality were assessed using four country- and gender-specific Cox proportional hazards models: (1) adjusted for age; (2) additionally adjusted for education; (3) additionally adjusted for marital status, occupational status, and smoking; and (4) additionally adjusted for self-rated health. The proportional hazard assumptions were verified using graphs of the log(-log(survival)) vs. the log of survival time. The timescale was the follow-up time in the study. Pooled analysis (of cohorts and genders) was not performed, since the interactions between the ATHLOS Healthy Aging Scale and country and gender, respectively, were statistically significant Statistical analyses were conducted using Stata version 14.1 (StataCorp LP, TX, USA), and IBM^®^ SPSS, with a *P*-value threshold of α < 0.05 for statistical significance.

## 3. Results

Table 1 shows distributions of age, the ATHLOS Healthy Aging Scale score, and follow-up time by country and gender. In both men and women, Czech participants tended to be older than Polish participants (57.4 vs. 56.9; *p* = 0.001 in women and 58.0 vs. 57.4; *p* < 0.001 in men). The mean ATHLOS Healthy Aging Scale score was higher for Czech women (50.3) than for Polish women (46.7; *p* < 0.001). No significant difference in the mean values of the ATHLOS Healthy Aging Scale score by country was found in men. The median values of follow-up time were approximately 5,500 days in Czechia and 5,000 days in Poland.

**Table 1:** Distribution of age, ATHLOS Healthy Aging Scale score, and follow-up time by country and gender.

	**Czechia**					**Poland**					***p***
	***x***	**SD**	**Me**	**Min**	**Max**	***x***	**SD**	**Me**	**Min**	**Max**	
**Women**	*N* = 4,570					*N* = 5,097					
Age (years)	57.4	7.1	58.0	44	72	56.9	7.0	56.0	45	70	0.001
ATHLOS Healthy Aging Scale (points)	50.3	8.6	50.3	25.8	63.9	47.5	9.0	46.7	25.8	63.9	<0.001
Follow-up time (days)			5,522	39	6,152			5,004	22	5,656	<0.001
**Men**	*N* = 3,948					*N* = 4,825					
Age (years)	58.0	7.2	58.0	44	72	57.4	7.0	57.0	45	70	<0.001
ATHLOS Healthy Aging Scale (points)	51.7	8.3	52.6	25.8	63.9	51.3	8.9	53.2	25.8	63.9	0.160
Follow-up time (days)			5,457	20	6,124			4,971	26	5,390	<0.001

*x*, mean; SD, standard deviation; Me, median; min, lowest value; max, highest value.

Table 2 presents the proportions of deaths by quintile of the ATHLOS Healthy Aging Scale score, country and sex. The differences in numbers within quintiles in Czechia and Poland were mainly a consequence of combining the two countries. In women, 656 and 674 deaths occurred, respectively, in Czechia and Poland. In men, there were 1,044 deaths in Czechia and 1,154 in Poland. The proportion of deaths in both genders and countries increased with the decreased quintile of the ATHLOS Healthy Aging Scale. Between-country differences in the proportion of deaths are largely dependent on the differences in the follow-up time. The distribution of covariates by country and gender is presented in Supplementary Table 1.

**Table 2:** Proportions of deaths by quintiles of ATHLOS Healthy Aging Scale score, country and gender.

	**Czechia**			**Poland**		
	***N***	**Died (** ***n*** **)**	**Died (%)**	***N***	**Died (** ***n*** **)**	**Died (%)**
**Women**						
Q1 ≤ 40.11	615	181	29	1,309	289	22
Q2 40.12–45.68	857	178	21	1,072	130	12
Q3 45.69–51.66	1,046	132	13	922	97	11
Q4 51.67–57.86	772	69	9	859	79	9
Q5 > 57.86	1,280	96	8	935	79	8
Total	4,570	656	14	5,097	674	13
**Men**						
Q1 ≤ 43.07	667	301	45	1,070	436	41
Q2 43.08–50.08	887	289	33	875	235	27
Q3 50.09–55.98	862	198	23	888	186	21
Q4 55.99–60.06	766	144	19	999	164	16
Q5 > 60.06	766	112	15	993	133	13
Total	3,948	1,044	26	4,825	1,154	24

The associations between quintiles of the ATHLOS Healthy Aging Scale score and all-cause mortality in women are shown in Table 3. For the Czech women, after adjusting for age, the risk of death for those in the lowest quintile of the ATHLOS Healthy Aging Scale (Q1) was approximately three times higher than those in the highest quintile (Q5). Significant increases in the risk of death were also observed in Q2 (HR = 2.04; 95% CI = 1.59–2.61) and Q3 (HR = 1.39; 95% CI = 1.07–1.80). A dose-response relationship between quintiles of the ATHLOS Healthy Aging Scale score and all-cause mortality was observed. Additional adjustment for education hardly changed the results. Further adjustments for marital status, occupational status, and smoking slightly attenuated the associations in the two lowest quintiles by ~12%. The association in the Q3 group became insignificant. In the final model, after additionally controlling for self-rated health, only a slight reduction in the estimates was observed. Ultimately, compared to women in the Q5 group, women in the Q1 group had over 2.5 times higher risk of death and women in the Q2 group had an ~80% higher risk of death. For the Polish women, significant unadjusted associations between quintiles of the ATHLOS Healthy Aging Scale score and all-cause mortality were observed only in those in the Q1 and Q2 groups. After controlling for age, compared to those in the Q5 group, women in the Q1 group had a nearly twice higher risk of death (HR = 1.96; 95% CI: 1.52–2.52). Further adjustments contributed to the attenuation of the magnitude of the observed association, with the greatest reduction observed in the final model. Eventually, compared to Polish women in the Q5 group, women in the Q1 group had a nearly 40% higher risk of death (HR = 1.38; 95% CI 1.05–1.81).

**Table 3:** Association between quintiles of ATHLOS Healthy Aging Scale score and all-cause mortality by country for the women.

**Women**	**Czechia**					**Poland**				
**ATHLOS Healthy Aging Scale**	**HR (95% CI)**	**HR (95% CI)**	**HR (95% CI)**	**HR (95% CI)**	**HR (95% CI)**	**HR (95% CI)**	**HR (95% CI)**	**HR (95% CI)**	**HR (95% CI)**	**HR (95% CI)**
Q5 > 57.86	1.00	1.00	1.00	1.00	1.00	1.00	1.00	1.00	1.00	1.00
Q4 51.67–57.86	1.21 (0.89–1.64)	1.08 (0.79–1.46)	1.06 (0.78–1.44)	1.05 (0.77–1.42)	1.04 (0.76–1.42)	1.10 (0.81–1.50)	1.01 (0.74–1.38)	1.00 (0.74–1.37)	0.97 (0.71–1.33)	0.92 (0.68–1.27)
Q3 45.69–51.66	1.82 (1.41–2.35)	1.39 (1.07–1.80)	1.36 (1.05–1.76)	1.27 (0.97–1.65)	1.23 (0.93–1.62)	1.24 (0.93–1.67)	1.04 (0.77–1.40)	1.02 (0.76–1.37)	0.99 (0.73–1.33)	0.87 (0.64–1.18)
Q2 40.12–45.68	3.01 (2.36–3.85)	2.04 (1.59–2.61)	1.96 (1.53–2.53)	1.82 (1.41–2.35)	1.78 (1.35–2.34)	1.45 (1.10–1.91)	1.06 (0.80–1.41)	1.03 (0.77–1.36)	0.98 (0.74–1.30)	0.83 (0.62–1.11)
Q1 ≤ 40.11	4.58 (3.59–5.85)	2.98 (2.32–3.82)	2.80 (2.18–3.61)	2.59 (2.00–3.36)	2.52 (1.90–3.36)	2.80 (2.18–3.58)	1.96 (1.52–2.52)	1.88 (1.46–2.42)	1.70 (1.32–2.20)	1.38 (1.05–1.81)
Age		1.10 (1.08–1.11)	1.10 (1.08–1.11)	1.09 (1.07–1.11)	1.09 (1.07–1.11)		1.09 (1.07–1.10)	1.09 (1.07–1.10)	1.07 (1.06–1.08)	1.07 (1.06–1.08)
University education			0.66 (0.47–0.93)	0.70 (0.49–0.98)	0.70 (0.50–0.99)			0.79 (0.65–0.95)	0.90 (0.74–1.10)	0.92 (0.75–1.12)
Marital status (married or cohabiting)				1.28 (1.10–1.50)	1.29 (1.10–1.51)				1.33 (1.14–1.56)	1.33 (1.14–1.55)
Occupational status (employed)				1.39 (1.11–1.75)	1.40 (1.11–1.76)				2.13 (1.68–2.71)	2.03 (1.60–2.59)
Smoking (ever smoked)				1.49 (1.27–1.74)	1.50 (1.28–1.75)				1.72 (1.48–2.01)	1.74 (1.48–2.03)
Self-rated health (good)					0.96 (0.78–1.17)					0.63 (0.50–0.79)

HR, hazard ratio; CI, confidence interval.

The associations between quintiles of the ATHLOS Healthy Aging Scale score and all-cause mortality in men are presented in Table 4. In the age-adjusted model for participants from Czechia, compared to men in the Q5 group, men in the Q3, Q2 and Q1 groups had a significantly higher risk of death by 34%, 88% and nearly 3 times, respectively. Further adjustment for covariates slightly attenuated the estimates. The greatest reduction in values of HR was observed in the Q1 group in the fully adjusted model. Nevertheless, the graded associations between the ATHLOS Healthy Aging Scale score and all-cause mortality remained significant. Compared to the Czech men in the Q5 group, men from the three lowest quintile groups had a higher risk of death by 20% (P3), 42% (P2) and 85% (P1), respectively. For the men from Poland, after adjusting for age, as well as for age and education, the results of the main associations were similar to those of the Czech men. Adjusting for more covariates contributed to a slight decrease in the estimates. In the final model, the association for Polish men in the Q3 group was insignificant. Ultimately, compared to Polish men in the Q5 group, the men in the two lowest quintile groups had a higher risk of all-cause mortality by 40% and over 2-fold, respectively.

**Table 4:** Association between quintiles of ATHLOS Healthy Aging Scale score and all-cause mortality by country for the men.

**Men**	**Czechia**					**Poland**				
**ATHLOS Healthy Aging Scale**	**HR (95% CI)**	**HR (95% CI)**	**HR (95% CI)**	**HR (95% CI)**	**HR (95% CI)**	**HR (95% CI)**	**HR (95% CI)**	**HR (95% CI)**	**HR (95% CI)**	**HR (95% CI)**
Q5 > 60.06	1.00	1.00	1.00	1.00	1.00	1.00	1.00	1.00	1.00	1.00
Q4 55.99–60.06	1.26 (0.99–1.60)	1.21 (0.95–1.54)	1.21 (0.95–1.54)	1.21 (0.94–1.55)	1.18 (0.92–1.51)	1.26 (1.001–1.57)	1.14 (0.91–1.43)	1.14 (0.91–1.43)	1.08 (0.86–1.36)	1.06 (0.84–1.33)
Q3 50.09–55.98	1.60 (1.28–2.00)	1.34 (1.07–1.69)	1.30 (1.03–1.63)	1.29 (1.02–1.62)	1.20 (0.94–1.52)	1.62 (1.30–2.02)	1.31 (1.05–1.63)	1.26 (1.01–1.58)	1.14 (0.91–1.43)	1.12 (0.89–1.40)
Q2 43.08–50.08	2.49 (2.02–3.08)	1.88 (1.52–2.33)	1.78 (1.44–2.21)	1.59 (1.27–1.99)	1.42 (1.12–1.79)	2.18 (1.76–2.70)	1.68 (1.36–2.08)	1.64 (1.33–2.04)	1.45 (1.17–1.80)	1.40 (1.12–1.75)
Q1 ≤ 43.07	3.81 (3.09–4.70)	2.83 (2.29–3.50)	2.68 (2.16–3.32)	2.19 (1.75–2.74)	1.85 (1.45–2.36)	3.67 (3.02–4.45)	2.66 (2.18–3.24)	2.46 (2.02–3.00)	2.14 (1.75–2.62)	2.04 (1.64–2.53)
Age		1.10 (1.09–1.11)	1.10 (1.09–1.11)	1.08 (1.07–1.10)	1.08 (1.07–1.10)		1.08 (1.07–1.09)	1.08 (1.07–1.09)	1.07 (1.06–1.08)	1.07 (1.06–1.08)
University education			0.75 (0.63–0.89)	0.86 (0.71–1.03)	0.87 (0.72–1.04)			0.65 (0.57–0.75)	0.75 (0.64–0.87)	0.76 (0.65–0.88)
Marital status (married or cohabiting)				1.58 (1.36–1.84)	1.58 (1.36–1.84)				1.74 (1.51–2.01)	1.75 (1.51–2.02)
Occupational status (employed)				1.60 (1.37–1.88)	1.57 (1.34–1.84)				1.29 (1.11–1.50)	1.28 (1.10–1.48)
Smoking (ever smoked)				1.87 (1.61–2.17)	1.88 (1.62–2.18)				1.97 (1.69–2.30)	1.97 (1.69–2.29)
Self-rated health (good)					0.75 (0.64–0.88)					0.91 (0.78–1.05)

HR, hazard ratio; CI, confidence interval.

## 4. Discussion

In a 14-year follow-up, the inverse associations between quintiles of the ATHLOS Healthy Aging Scale and all-cause mortality were found in both men and women in each country. These associations were independent of age, education, marital status, occupational status, smoking and self-rated health. For the Polish women, the strength of the association was lower than for the Czech women and lower than for the men from both countries. The pattern of association may depend to some extent on the baseline distribution of the ATHLOS Healthy Aging Scale scores, which for the Polish women was the lowest.

The results of our study are consistent with the previous analysis of data from England (9). Older people's life expectancy does not depend on health status at older age alone but rather depends on the long-term interaction between individuals' intrinsic capacities and the environment they live in (6). It is therefore an important observation that the ATHLOS Healthy Aging Scale had a similar ability to predict mortality in both Western European and Central and Eastern European countries, i.e., populations exposed to different social contexts during their lives.

Our results are consistent with the findings of other studies on particular domains such as mobility, cognition, and activities of daily living. For physical functioning, including locomotion and activities of daily living, several studies found a negative association between objective measures of physical functioning and all-cause mortality. A recent meta-analysis concluded that physical capability is a predictor of all-cause mortality in older adults (13). Further studies have reinforced the conclusion that limitations in physical functioning, defined as having the inability to complete at least one of the performance-based tests (grip strength, timed walk, chair stands, and peak expiratory flow), are associated with approximately twice higher risks of subsequent 4-year mortality (14). For cognitive functions, there is also well-established evidence indicating the inverse relationship between cognitive functions (15–17) and psychosocial factors with mortality (18, 19).

Studies on the relationships between single exposures related to daily functioning and mortality are numerous, and they have provided clear evidence of an adverse relationship between functional limitations and the risk of death in older people. However, the focus on single exposures (of functional domains) might be somewhat detached from “normal life”. For example, some older people with physical limitations may still be able to achieve healthy aging if they maintain good levels of cognitive performance and psychosocial wellbeing in their social and natural environment (2, 20). We confirmed the relation between morality and the ATHLOS Healthy Aging Scale which combines both intrinsic capacities and functional abilities in different domains. The advantage of this assessment is that it reflects an overall functioning comprehensively.

Compared to the commonly used frailty index, the ATHLOS Healthy Aging Scale includes more information on functional capabilities. The frailty index focuses more on age-related health deficits. It is considered a proxy measure of biological aging (21, 22) and was also found to be associated with elevated mortality risk (23).

The strengths of our study include using two population-based representative samples to investigate an important yet under-researched epidemiological question in a geographical region with a high risk of total mortality since the late 1990s. Using cohort data, we examined time trends over a period of up to 14 years after baseline data collection. We confirmed a good predictive performance of the ATHLOS Healthy Aging Scale for all-cause mortality in a different socio-economic context from Western Europe. Our results can contribute to a wider acceptance of this standardized healthy aging index.

There are also some limitations that should be considered. First, the ATHLOS Healthy Aging Scale has some limitations in the interpretation of the results, which has been widely discussed by Sanchez-Niubo (8). One important limitation is that data used for the development of the ATHLOS Healthy Aging Scale were among community dwellings. Therefore, this scale might under-represent older people with greater dependency, such as those living in nursing homes, other institutionalized persons, or those with greater cognitive impairments.

Second, the procedure for calculating the score of the ATHLOS Healthy Aging Scale might be difficult to replicate on a smaller scale. The large number of variables used to calculate an individual's ATHLOS Healthy Aging Scale score may not always be available, and this may affect the accuracy of the Healthy Aging Scale estimates. Given the number of measurements required, the tool's usefulness may be limited for large population-based assessments but be less useful for everyday practice. Finally, in the Czech and Polish HAPIEE samples, the response rate was ~ 60% and it is known that less healthy individuals are less likely to participate in studies such as the HAPIEE study (24). Furthermore, participants who had missing data for the ATHLOS Healthy Aging Scale, mortality information, and covariates were excluded from the analyses. Thus, the studied groups may not be fully representative of the target populations from Czechia and Poland and the associations found in the healthier parts of these populations might be underestimated.

Nevertheless, the ATHLOS Healthy Aging Scale has shifted the approach to measuring healthy aging from “being away from diseases only” to “considering the interaction between persons' intrinsic capacities and the environments they live in”. Demonstrating the significant relationship between the ATHLOS Healthy Aging Scale and mortality risk also opens the door to intervention studies that may target strengthening the functional ability in older people by improving their intrinsic capacities and creating aging-friendly environments to enable good functioning for individuals with some functional limitations.

## 5. Conclusion

In conclusion, the ATHLOS Healthy Aging Scale was a good predictor of all-cause mortality in urban populations of Poland and Czechia. This composite indicator of intrinsic capacity and functional ability may be an important contribution to a better assessment of healthy aging.

## Data availability statement

The raw data supporting the conclusions of this article will be made available by the authors, without undue reservation.

## Ethics statement

The studies involving human participants were reviewed and approved by National Institute of Public Health Ethics Committee (Prague, the Czech Republic) and Jagellonian University Medical College Bioethics Committee (Krakow, Poland). The patients/participants provided their written informed consent to participate in this study.

## Author contributions

AP, JA-M, MB, HP, AS-N, and JH contributed to the conception and design of the study. MK, KS, and MP organized the database. KS and MP performed the statistical analysis. MK wrote the first draft of the manuscript. WL and US wrote sections of the manuscript. All authors contributed to the revision of the manuscript and read and approved the submitted version.
