# Supplementary material for: ATHLOS Healthy Aging Scale score as the predictor of all-cause mortality in Poland and Czechia
Source: Front Public Health. 2023 Mar 16;11:1114497. doi: 10.3389/fpubh.2023.1114497 (PMC10061126; doi:10.3389/fpubh.2023.1114497)
Supplement: Supplementary file 1 [file Table_1.docx]

| Supplementary table 1. Distribution of marital status, education, occupational status, smoking and self-rated health by country and sex | | | | | |
| --- | --- | --- | --- | --- | --- |
|  | Czech Republic | | Poland | |  |
|  | n | % | n | % | p |
| **Women** | N=4,570 | | N=5,097 | |  |
| Marital status (married or cohabiting) | 3,119 | 68 | 3,406 | 67 | 0.141 |
| University education | 459 | 10 | 1,385 | 27 | <0.001 |
| Occupational status (employed) | 2,158 | 47 | 2,003 | 39 | <0.001 |
| Smoking (ever smoked) | 2,076 | 45 | 2,530 | 50 | <0.001 |
| Self rated health (good) | 1,860 | 41 | 1,625 | 32 | <0.001 |
| **Men** | N=3,948 | | N=4,825 | |  |
| Marital status (married or cohabiting) | 3,326 | 84 | 4,187 | 87 | 0.001 |
| University education | 725 | 18 | 1,459 | 30 | <0.001 |
| Occupational status (employed) | 2,291 | 58 | 2,327 | 48 | <0.001 |
| Smoking (ever smoked) | 2,689 | 68 | 3,486 | 72 | <0.001 |
| Self rated health (good) | 1,570 | 40 | 1,949 | 40 | 0.550 |
